# Anti-Inflammatory Effects of Formononetin 7-*O*-phosphate, a Novel Biorenovation Product, on LPS-Stimulated RAW 264.7 Macrophage Cells

**DOI:** 10.3390/molecules24213910

**Published:** 2019-10-30

**Authors:** Min-Seon Kim, Jin-Soo Park, You Chul Chung, Sungchan Jang, Chang-Gu Hyun, Seung-Young Kim

**Affiliations:** 1 Department of Pharmaceutical Engineering & Biotechnology, Sunmoon University, Chungnam 31460, Korea; nari7040@gmail.com (M.-S.K.); biochem1004@gmail.com (S.J.); 2 Natural Product Informatics Research Center, KIST Gangneung Institute of Natural Products, Korea Institute of Science and Technology (KIST), 679, Saimdang-ro 25451, Korea; jinsoopark@kist.re.kr; 3 Department of Chemistry and Cosmetics, Jeju National University, Jeju 63243, Korea; jyc8385@hanmail.net (Y.C.C.); cghyun@jejunu.ac.kr (C.-G.H.)

**Keywords:** biorenovation, formononetin 7-*O*-phosphate, anti-inflammatory, MAPK pathway, NF-κB pathway

## Abstract

Biorenovation is a microbial enzyme-catalyzed structural modification of organic compounds with the potential benefits of reduced toxicity and improved biological properties relative to their precursor compounds. In this study, we synthesized a novel compound verified as formononetin 7-*O*-phosphate (FMP) from formononetin (FM) using microbial biotransformation. We further compared the anti-inflammatory properties of FMP to FM in lipopolysaccharide (LPS)-treated RAW264.7 macrophage cells. We observed that cell viabilities and inhibitory effects on LPS-induced nitric oxide (NO) production were greater in FMP-treated RAW 264.7 cells than in their FM-treated counterparts. In addition, FMP treatment suppressed the production of proinflammatory cytokines such as prostaglandin-E_2_ (PGE_2_), interleukin-6 (IL-6), and interleukin-1β (IL-1β) in a dose-dependent manner and concomitantly decreased the mRNA expression of inducible NO synthase (iNOS) and cyclooxygenase-2 (COX-2). We also found that FMP exerted its anti-inflammatory effects through the downregulation of the extracellular signal-regulated kinase (ERK), c-Jun *N*-terminal kinase (JNK), and nuclear factor kappa B (NF-κB) signaling pathways. In conclusion, we generated a novel anti-inflammatory compound using biorenovation and demonstrated its efficacy in cell-based in vitro assays.

## 1. Introduction

Inflammation is an early host immune reaction mediated by cytokines secreted from immune cells [1,2]. This response plays a key role in the development of various human chronic diseases, including bronchitis, cancer, diabetes, and rheumatoid arthritis [3]. Macrophages, which play a major role in the immune response, perform an important role in the initiation, maintenance, and resolution of inflammation [4]. Macrophages are activated by stimuli such as interferon-γ (IFN-γ), bacterial lipopolysaccharides (LPS), proinflammatory cytokines (including tumor necrosis factor- α (TNF-α), interleukin-6 (IL-6), and interleukin-1β (IL-1β)), extracellular matrix proteins, and other chemical mediators [5]. Upon activation by these stimuli, macrophages produce numerous proinflammatory mediators, such as nitric oxide (NO), prostaglandin-E2 (PGE2), and TNF-α, to promote inflammatory responses [6]. NO is a key signaling molecule in a number of biological processes, including vasodilation, neurotransmission, the inhibition of platelet aggregation, and the immune response. NO is generated through the oxidation of L-arginine, which is catalyzed by nitric oxide synthase (NOS) enzymes [7,8,9]. The overproduction of NO by inducible nitric oxide synthase (iNOS) forms reactive nitrogen species, resulting in cell death in surrounding tissues and the disruption of tissue homeostasis [10,11].

PGE_2_, another mediator of inflammation, is known to induce cancer by activating angiogenesis [12,13,14]. Cyclooxygenase (COX) is a key enzyme in the biosynthetic pathway of PGE_2_, with two isozymes, COX-1 and COX-2. COX-2 can be upregulated by various inflammatory cytokines. In contrast, COX-1 is constitutively expressed in most cells at an essentially constant level and plays an important role in housekeeping functions [15,16,17].

The LPS stimulation of macrophages activates several intracellular signaling pathways that include the IκB kinase (IKK)–nuclear factor kappa B (NF-κB) pathway and three classical mitogen-activated protein kinase (MAPK) pathways: extracellular signal-regulated kinase (ERK), c-Jun N-terminal kinase (JNK), and p38 [18,19,20]. The activation of NF-κB, when induced by LPS, involves the phosphorylation of IKK, leading to the subsequent ubiquitination and degradation of IκBα and the translocation of NF-κB into the nucleus. The MAPK pathways modulate inflammatory gene transcription through the phosphorylation of the ERK, JNK, and p38 proteins [21,22]. The activation of these signaling pathways in turn activates a variety of transcription factors that control the expression of genes involved in inflammation, including iNOS and COX-2 [23]. Accordingly, MAPKs and NF-κB are important targets for anti-inflammatory molecules, and many putative anti-inflammatory therapies are based on the inhibition of their activity.

Flavonoids are a broad class of low-molecular-weight secondary plant phenolic compounds characterized by a flavan nucleus [24]. Widely distributed in the leaves, seeds, bark, and flowers of plants, over 4000 flavonoids have been identified to date [25]. Among them, formononetin (FM, biochanin B) is an *O*-methylated isoflavone phytoestrogen from the root of *Astragalus membranaceus* [26] that is reported to have antibreast cancer [27], estrogenic [28], and antihypertensive [29] effects.

To study the development of new anti-inflammatory drug sources, FM was converted using a biorenovation technique described previously [30,31]. Biorenovation is a method of producing various derivatives from precursors using the biocatalytic reactions of microorganisms. In this study, *Bacillus amyloliquefaciens* KCTC 13,588 was used as a biocatalyst to produce FM derivatives. The aim of this study was to investigate the anti-inflammatory activity of biorenovated FM and to establish its functional properties by examining the expression levels of various factors.

## 2. Results

### 2.1. Analysis and Identification of the Biorenovation Product of Formononetin

**Figure 1:**
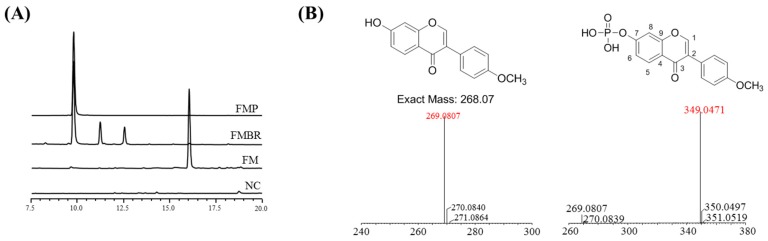
(**A**) HPLC analysis of formononetin (FM), a formononetin biorenovation product (FMBR), a biorenovation negative control (NC), and formononetin 7-*O*-phosphate (FMP); (**B**) structure and mass analysis of FM and FMP.

In this study, *Bacillus amyloliquefaciens* KCTC 13,588 was used for the production of biorenovation derivatives of FM. The reaction products were detected by HPLC analysis. Three peaks distinct from those of the standard compound were found in the culture supernatant (Figure 1A). Preparative HPLC was used to purify the compound (compound 1) responsible for the largest of these peaks. Electrospray ionization–mass spectrometry (ESI/MS) analysis was then performed to determine if the compound was a derivative of FM. The mass spectrum of compound 1 showed a representative peak at *m*/*z* 269 corresponding to the characteristic fragmentation of FM. This result suggested that compound 1 was a derivative of FM. The molecular formula C_16_H_13_O_7_P, which was determined by high-resolution ESI/MS (HR-ESI/MS), suggested that this compound was a phosphorylated form of FM. For structural identification, compound 1 was analyzed by 1D and 2D NMR, including ^1^ H NMR, ^13^C NMR, HSQC, and HMBC spectroscopy. Proton signals (7.29 ppm for H-6 and 7.41 ppm for H-8) of compound 1 were downfield-shifted compared to those of FM (6.94 ppm for H-6 and 6.86 ppm for H-8), suggesting the presence of a phosphate group at 7-OH. Furthermore, the phosphorylation at 7-OH was confirmed by split carbon signals of C-6 and C-8 due to C–P coupling (*J_C_*–*_P_* = 5.5 Hz). To our knowledge, this structure, formononetin 7-*O*-phosphate (FMP), has not been reported to date (Figure 1B).

### 2.2. NMR Results

Formononetin 7-*O*-phosphate (FMP, **1**): ^1^H NMR (DMSO-d_6_, 500 MHz): δ 8.43 (1H, s, H-2), 8.08 (1H, d, *J* = 8.8 Hz, H-5), 7.51 (2H, m, H-2′, H-6′), 7.41 (1H, d, *J* = 2.2 Hz, H-8), 7.29 (1H, dd, *J* = 8.8, 2.2 Hz, H-6), 6.99 (2H, m, H-3′, H-5′), 3.78 (3H, s, OCH_3_). ^13^C NMR (DMSO-*d_6_*, 125 MHz): δ 175.14 (C-4), 159.46 (C-4′), 156.86 (C-8a), 156.75 (C-7), 154.35 (C-2), 130.53 (C-2′, C-6′), 127.49 (C-5), 124.32 (C-3), 123.85 (C-1′), 120.10 (C-4a), 118.84 (C-6, d, *J* = 5.5 Hz), 114.06 (C-3′, C-5′), 108.44 (C-8, d, *J* = 5.0 Hz), 55.59 (OCH_3_). HR-ESI/MS: *m*/*z* [M + H] 349.0471, calcd 349.0477 (see Supplementary Materials).

### 2.3. Cytotoxic Effects of Compounds on RAW 264.7 Cells

**Figure 2:**
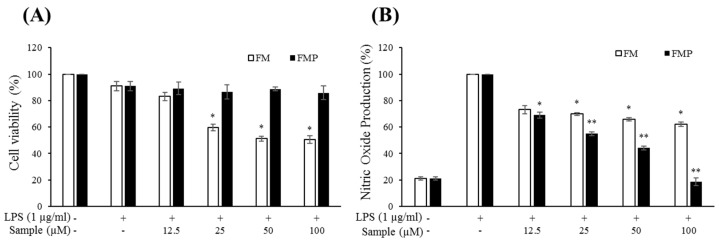
Effects on cell viability and nitric oxide production by FM and FMP in lipopolysaccharide (LPS)-stimulated RAW 264.7 cells. (**A**) Cell viability was assessed in cells stimulated with LPS (1 µg/mL) in the presence of FM or FMP for 24 h; (**B**) nitric oxide production was determined using the Griess reagent method. The data represent the mean ± SD of triplicate experiments. * *p* < 0.05, ** *p* < 0.01 versus LPS alone.

RAW 264.7 macrophage cells were treated with varying concentrations of FM or FMP (12.5, 25, 50, and 100 µM) with or without LPS (1 µg/mL) for 24 h. An MTT (3-(4,5-dimethylthiazol-2-yl)-2,5-diphenyltetrazolium bromide) assay showed that cell viability decreased by 42%, 48%, and 50% in cells treated with 25, 50, and 100 µM FM, respectively, but that FMP was nontoxic to RAW 264.7 cells at these concentrations (Figure 2A). These results indicate that the changes made through biorenovation contributed to the improvement of cell viability.

### 2.4. Production of NO and PGE_2_

**Figure 3:**
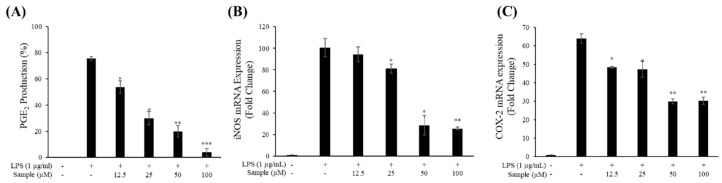
Effects of FMP on prostaglandin-E_2_ (PGE_2_) production and mRNA levels of inducible-nitric oxide synthase (iNOS) and cyclooxygenase-2 (COX-2) in LPS-stimulated RAW 264.7 cells. (**A**) The production of PGE_2_ was assayed in the culture medium of cells stimulated with LPS (1 μg/mL) for 24 h in the presence of FMP (12.5, 25, 50, and 100 µM) by ELISA; (**B**,**C**) mRNA levels of iNOS and COX-2 were determined by qRT-PCR. The data represent the mean ± SD of triplicate experiments. * *p* < 0.05, ** *p* < 0.01, *** *p* < 0.005 versus LPS alone.

The effects of FM and FMP on NO production were measured in cells treated with various concentrations of FM and FMP for 24 h. LPS (1 μg/mL) was used as a negative control. As shown in Figure 2B, FM treatment decreased NO production compared to the group treated with LPS alone. However, this result was accompanied by notable decreases in cell viability (Figure 2A). FMP, however, reduced NO production in a dose-dependent manner without signs of cytotoxicity. Overall, FMP displayed a greater inhibition of NO production than FM did. Thus, FMP treatment was chosen to measure PGE_2_ concentrations. As a result, PGE_2_ production decreased 30%, 50%, 60%, and 90% in cells treated with 12.5, 25, 50, and 100 µM FMP, respectively (Figure 3A).

### 2.5. mRNA Levels of iNOS and COX-2

To determine whether the inhibitory effect of FMP on NO and PGE_2_ production was due to the suppression of iNOS and COX-2 expression, the mRNA expression of these enzymes was measured. FMP significantly reduced the expression of iNOS at concentrations ranging from 25 to 100 µM (Figure 3B) and the expression of COX-2 at concentrations ranging from 12.5 to 100 µM (Figure 3C) relative to the group treated with LPS alone. These results indicate that the reduction of iNOS and COX-2 mRNA levels was key to the decreased expression of NO and PGE_2_.

### 2.6. Production of Proinflammatory Cytokines

**Figure 4:**
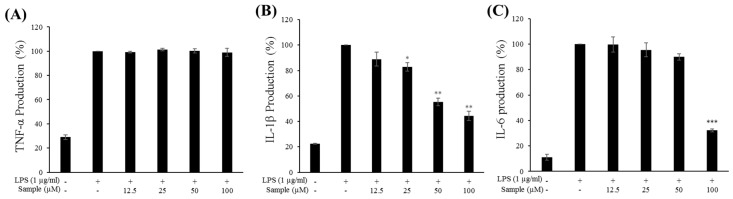
Effect of FMP on (**A**) tumor necrosis factor-α (TNF-α), (**B**) interleukin-1β (IL-1β), and (**C**) interleukin-6 (IL-6) production in LPS-stimulated RAW 264.7 cells. Cells were stimulated with 1 µg/mL of LPS only or with LPS plus varying concentrations (12.5, 25, 50, and 100 µg/mL) of FMP for 24 h. Protein production was determined through an ELISA. The data represent the mean ± SD of triplicate experiments. * *p* < 0.05, ** *p* < 0.01, *** *p* < 0.005 versus LPS alone.

An ELISA was performed to assess the effects of FMP on the production of proinflammatory cytokines (TNF-α, IL-1β, and IL-6) in RAW 264.7 cells. As shown in Figure 4B,C, FMP treatment at the indicated concentrations reduced IL-1β and IL-6 expression in a dose-dependent manner. Notably, treatment with FMP at 100 μM inhibited IL-1β and IL-6 expression by 50% and 70%, respectively, compared to the group treated with LPS alone. However, treatment with FMP did not affect TNF-α expression, indicating that TNF-α may not be involved in mediating the anti-inflammatory effects of FMP (Figure 4A).

### 2.7. Expression of MAPK and NF-κB Pathways

**Figure 5:**
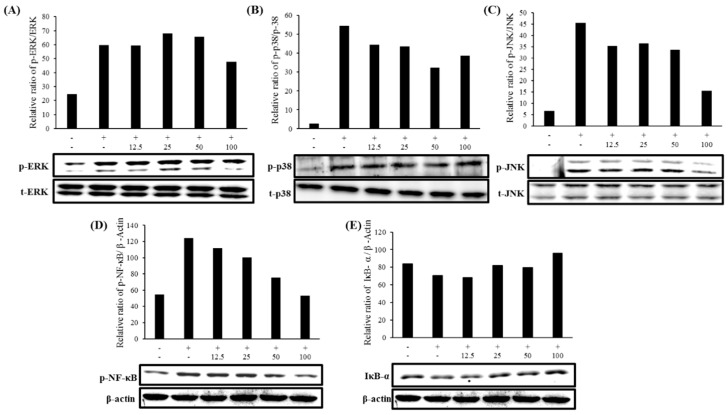
Effect of FMP on the protein levels of (**A**) extracellular signal-regulated kinase (ERK), (**B**) p38, (**C**) c-Jun N-terminal kinase (JNK), (**D**) nuclear factor kappa B (NF-κB), and (**E**) IκB kinase in LPS-stimulated RAW 264.7 cells. Cells (1.5 × 10^5^ cell/mL) were stimulated with LPS (1 µg/mL) in the presence of FMP (12.5, 25, 50, and 100 µM) for 30 min. Whole-cell lysates (30 µg) were prepared, the protein level was subjected to 10% SDS-PAGE, and the expression of MAPK, NF-κB and β-actin was determined by western blotting.

To study the effects of FMP on inflammatory signaling pathways in LPS-stimulated RAW 264.7 macrophage cells, MAPK and NF-κB signaling pathway proteins were measured. As shown in Figure 5C, the phosphorylation of JNK was significantly diminished by FMP. Both ERK and p38 also exhibited slightly decreased phosphorylation at the highest concentration of FMP (Figure 5A,B). These results also suggest that FMP treatment may affect the NF-κB signaling pathway by increasing the expression level of IκB-α and decreasing the phosphorylation level of NF-κB (Figure 5D,E). These results indicate that FMP inhibited the expression of proinflammatory cytokines and inflammatory mediators through the MAPK and NF-κB signaling pathways.

## 3. Discussion

To develop a novel anti-inflammatory agent, we employed a biorenovation process using FM as a substrate. We identified this new compound as FMP using MS and NMR.

Macrophages are known to play a critical role in acute and chronic inflammatory responses through the production of proinflammatory factors such as NO, PGE_2_, and cytokines, including TNF-α, IL-1β, and IL-6. Exposure to high levels of NO can induce an innate immune response and cause tissue damage or cell injury [32].

In this study, FMP was shown to exhibit lower cytotoxicity in macrophages relative to FM, indicating that cell viability was improved by the biorenovation reaction. Therefore, further research was conducted examining the anti-inflammatory activity of FMP. Treatment with FMP reduced the secretion of NO and PGE_2_ and the mRNA expression of their respective enzymes, iNOS and COX-2, in a dose-dependent manner. These results suggest that the inhibition of NO and PGE_2_ production seen in cells was due to the downregulation of mRNA expressions of iNOS and COX-2.

The proinflammatory cytokines TNF-α, IL-6, and IL-1β cause tissue damage and play a key role in mediating various inflammatory diseases [6]. FMP decreased the secretion of IL-6 and IL-1β but had no effect on TNF-α production. Therefore, we suggest that FMP exerted its anti-inflammatory effects through the reduced production of IL-6 and IL-1β.

We also investigated whether or not the anti-inflammatory effects of FMP were mediated through the MAPK and NF-κB signaling pathways via the effects of FMP on the LPS-induced phosphorylation of upstream kinases. FMP treatment lowered the upregulated expression levels of phosphorylated ERK, JNK, p38, NF-kB, and IκB seen in the treatment group to the level of the untreated control group. Thus, the decreased production of iNOS, COX-2, and proinflammatory cytokines was concluded to be due to the modulation of the MAPK and NF-kB signaling pathways.

Additionally, our previous research [30,31] indicated that the two additional compounds observed in the HPLC chromatogram of FMBR (Figure 1A) were ononin (formononetin 7-*O*-glucoside, C_22_H_22_O_9_) and succinyl-ononin (C_26_H_26_O_12_). The macrophage-mediated anti-inflammatory effects of ononin were studied by L Dong et al. [33], with results similar to those of FMP in this study. However, FMP inhibited the production of PGE_2_ more effectively than ononin, suggesting that the compounds act by different mechanisms. Further investigation into the anti-inflammatory activity of succinyl-ononin is needed before its effects can be compared to those of FMP.

With interest in research on the health-promoting effects of flavonoid compounds growing [34], we have shown here that a biorenovation product can exhibit stronger anti-inflammatory properties than its substrate. As expected, the anti-inflammatory activity of FMP was regulated through the MAPK and NF-kB pathways, and its activity was higher than FM. Our results suggest that formononetin 7-*O*-phosphate, a novel compound synthesized by biorenovation, may be a promising candidate in the development of new anti-inflammatory drugs.

## 4. Materials and Methods

### 4.1. Reagents and Bacterial Strain Used in Biorenovation

Formononetin was purchased from Selleck Chemicals (Houston, TX, USA). Nutrient medium components were purchased from Difco (Baltimore, MD, USA). The microorganism *B. amyloliquefaciens* KCTC 13,588 was purchased from KCTC (Korean Collection for Type Cultures, Seoul, Republic of Korea). All reagents for RAW 264.7 cell culture were purchased from Bio-rad (Hercules, CA, USA) or Cell Signaling Technology (Danvers, MA, USA).

### 4.2. Biorenovation of Formononetin

For use as an inoculum, *B. amyloliquefaciens* KCTC 13,588 was cultured by placing colonies from agar plates in 4 mL of nutrient broth (3 g/L of beef extract (ThermoFisher, Waltham, MA, USA) and peptone (ThermoFisher) 5 g/L) in a culturing tube, and 0.2% of the inoculum of *B. amyloliquefaciens* KCTC 13,588 was added to a 500-mL flask containing 100 mL of nutrient medium and cultured at 37 °C and 200 rpm for 18 h. Culture broth was centrifuged at 5000 rpm for 15 min, and the supernatant was discarded. The remaining cells were washed with phosphate glycerin buffer (PG buffer) containing 2% *v*/*v* glycerin in 50 mM of sodium phosphate at pH 7.2. After adding 5 mL of PG buffer, cells were resuspended through vortexing and centrifuging at 5000 rpm for 5 min. After discarding the supernatant, the washing was repeated. Formononetin was added to a final concentration of 4 mg/mL. Media were further incubated at 30 °C and 200 rpm for 48 h. After centrifugation at 5000 rpm for 10 min, the supernatant was concentrated using an evaporator.

### 4.3. HPLC Analysis and Purification of Formononetin Biorenovation Product

For HPLC analysis, a Shimadzu SpectroMonitor 3200 digital UV-Vis detector equipped with a Shim-pack GIS 0.5-mm ODS C18 column (250 × 4.6 mm id) was used (Shimadzu, Kyoto, Japan). The mobile phase consisted of water containing 0.1% *v*/*v* trifluoroacetic acid (TFA) (solvent A) and acetonitrile (solvent B). A gradient method was used with a flow rate of 1 mL/min of solvent B, which was increased from 10% to 100% over 30 min, maintained for 5 min, and then reduced to 10% over 3 min and maintained for 5 min.

### 4.4. LCMS and NMR Analysis of Formononetin Biorenovation Product

High-resolution quadrupole-time-of-flight electrospray ionization–mass spectrometry (HR-QTOF ESI/MS) analysis was performed in positive ion mode using an ACQUITY UPLC system coupled with a SYNAPT G2-Si column (Waters Corporation, Milford, MA, USA). NMR spectra were measured using a VNMRS 500 NMR spectrometer (Agilent Technology, Santa Clara, CA, USA), and residual solvent peaks (DMSO-d_6_ = δ_H_ 2.50) of deuterated NMR solvents (Sigma-Aldrich, St. Louis, MA, USA) were used as reference peaks.

### 4.5. Cell Culture and Viability Assay

RAW 264.7 macrophage cells were acquired from the Korean Cell Line Bank (KCLB, Seoul, Korea). Cells were cultured in DMEM supplemented with 10% heat-inactivated FBS with 1% penicillin and streptomycin and placed in a humidified incubator in a 5% CO_2_ atmosphere at 37 °C. At 80%–90% confluence, cells were plated at a density of 1.5 × 10^5^ cells/well in 24-well plates and incubated for 24 h. Cells were treated with varying concentrations of either FM or FMP (12.5, 25, 50, and 100 µM) with or without LPS (1 µg/mL) for 24 h. Cell viability was measured using a 3-(4,5-dimethylthiazol-2-yl)-2,5-diphenyltetrazolium bromide (MTT) assay [35]. MTT reagent (Sigma-Aldrich, St. Louis, MA, USA) was added at a concentration of 5 mg/mL to each well, and cells were then incubated for 3h. Subsequently, formazan crystals were dissolved in DMSO, and absorbance at 570 nm was read using a microplate reader (Spectrophotometer, ThermoFisher).

### 4.6. Determination of NO and PGE_2_ Production

RAW 264.7 cells were plated at a density of 1.5 × 10^5^ cells/well in 24-well plates and incubated for 24 h. Cells were treated with varying concentrations of either FM or FMP (12.5, 25, 50, and 100 µM) (then with or without LPS (1 µg/mL)) for 24 h. Cell culture supernatant (100 µL) was mixed with Griess reagent (Sigma-Aldrich, St. Louis, MA, USA) (100 µL), and absorbance was determined at 540 nm to measure NO production. The concentration of PGE_2_ in the culture supernatant of cells treated with FMP 12.5, 25, 50, and 100 µM was detected using a PGE_2_ ELISA Kit (Mouse PGE_2_, R&D Systems, MN, USA).

### 4.7. Determination of TNF-α, IL-1β, and IL-6 Production

RAW 264.7 cells were plated at a density of 1.5 × 10^5^ cells/well in 24-well plates and incubated for 24 h. Cells were treated with varying concentrations of FMP (12.5, 25, 50, and 100 µM) (then with or without LPS (1 µg/mL)) for 24 h. The concentrations of proinflammatory cytokines (TNF-α, IL-1β, and IL-6) in culture supernatant were determined using ELISA kits (Mouse TNF alpha ELISA Kit, Invitrogen, Carlsbad, CA, USA; Mouse IL-6 ELISA Kit, BD, Franklin Lakes, NJ, USA; Mouse IL-1β/IL-1F2, R&D Systems, Minneapolis, MN, USA). 

### 4.8. Quantitative Reverse-Transcription Polymerase Chain Reaction (qRT-PCR) Analysis

The total RNA obtained from RAW264.7 cells was isolated using an RNA extraction kit (RNeasy Mini Kit, QIAGEN, Hilden, Germany). Total RNA (1 µg) was reverse-transcribed using a cDNA synthesis kit (PrimeScript 1st strand cDNA Synthesis Kit, TaKaRa, Kyoto, Japan), and qRT-PCR was performed using an SYBR Mixture (TB Green Premix Ex Taq Ⅱ, TaKaRa, Kyoto, Japan). The primers used were as follows: iNOS-(F)AATGGCAACATCAGGTCGGCCATCACT; iNOS-(R) GCTGTGGTCACAGAAGTCTCGAACTC; COX-2-(F)GGAGAGACTATCAAGATAGT; COX-2-(R)ATGGTCAGTAGACTTTTACA; GAPDH(Glyceraldehyde 3-phosphate dehydrogenase)-(R)GGTTTCTCCAGGCGGCA; and GAPDH-(F)GGCATGGCCTTCCGTGT.

### 4.9. Western Blot Analysis

The total protein was extracted from the cells using RIPA buffer (Bio-red, Hercules, CA, USA) and measured with a Bradford assay kit (Pierce BCA Protein Assay Kit, Thermo Scientific, Waltham, MA, USA). Then, 20 µg of proteins were separated by 10% SDS-PAGE gel and transferred onto polyvinylidene difluoride (PVDF) membranes (Bio-red) at 250 V and for 1 h. Membranes were blocked with 5% skim milk at 1 h and incubated with primary Phospho-p44/42 MAPK (Erk1/2)(Thr202/Tyr204) Antibody (1:1000), Phospho-p38 MAP Kinase (Thr180/Tyr182) Antibody (1:500), Phospho-SAPK/JNK (Thr183/Tyr185) Antibody (1:500), p44/42 MAPK (Erk1/2) antibody (1:1000), p38 MAPK Antibody (1:500), SAPK/JNK Antibody (1:500), Phospho-NF-kB p65 (Ser536)(93H1) Rabbit mAb (1:500), and IκBα (L35A5) Mouse mAb Amino-Terminal Antigen (1:500) (Cell Signaling) at 4 °C overnight, followed by incubation with secondary antibody (1:10,000) (HRP Anti-Rabbit IgG (H&L), Rockland Immunochemicals, Inc., USA) at 25 °C for 1 h. Proteins were detected using an ECL kit (Bio-red, Hercules, California, USA) and measured using an Image reader (LAS-4000, FUJIFILM, Tokyo, Japan).

### 4.10. Statistical Analysis

Results are expressed in terms of mean ± SD. The statistical significance of the differences was evaluated using Student’s *t*-test for the data acquired.

## Supplementary Materials

The following are available online, Figure S1: 1H NMR of formononetin 7-*O*-phosphate (FMP, 1), Figure S2: 13C NMR of formononetin 7-*O*-phosphate (FMP, 1), Figure S3: HSQC correlation of formononetin 7-*O*-phosphate (FMP, 1), Figure S4: HMBC correlation of formononetin 7-*O*-phosphate (FMP, 1), Figure S5: 1H NMR of formononetin 7-*O*-glucoside.
