# Supplementary material for: Anti-Inflammatory Effects of Formononetin 7-O-phosphate, a Novel Biorenovation Product, on LPS-Stimulated RAW 264.7 Macrophage Cells
Source: Molecules. 2019 Oct 30;24(21):3910. doi: 10.3390/molecules24213910 (PMC6864718; doi:10.3390/molecules24213910)
Supplement: Supplementary file 1 [file molecules-24-03910-s001.pdf]

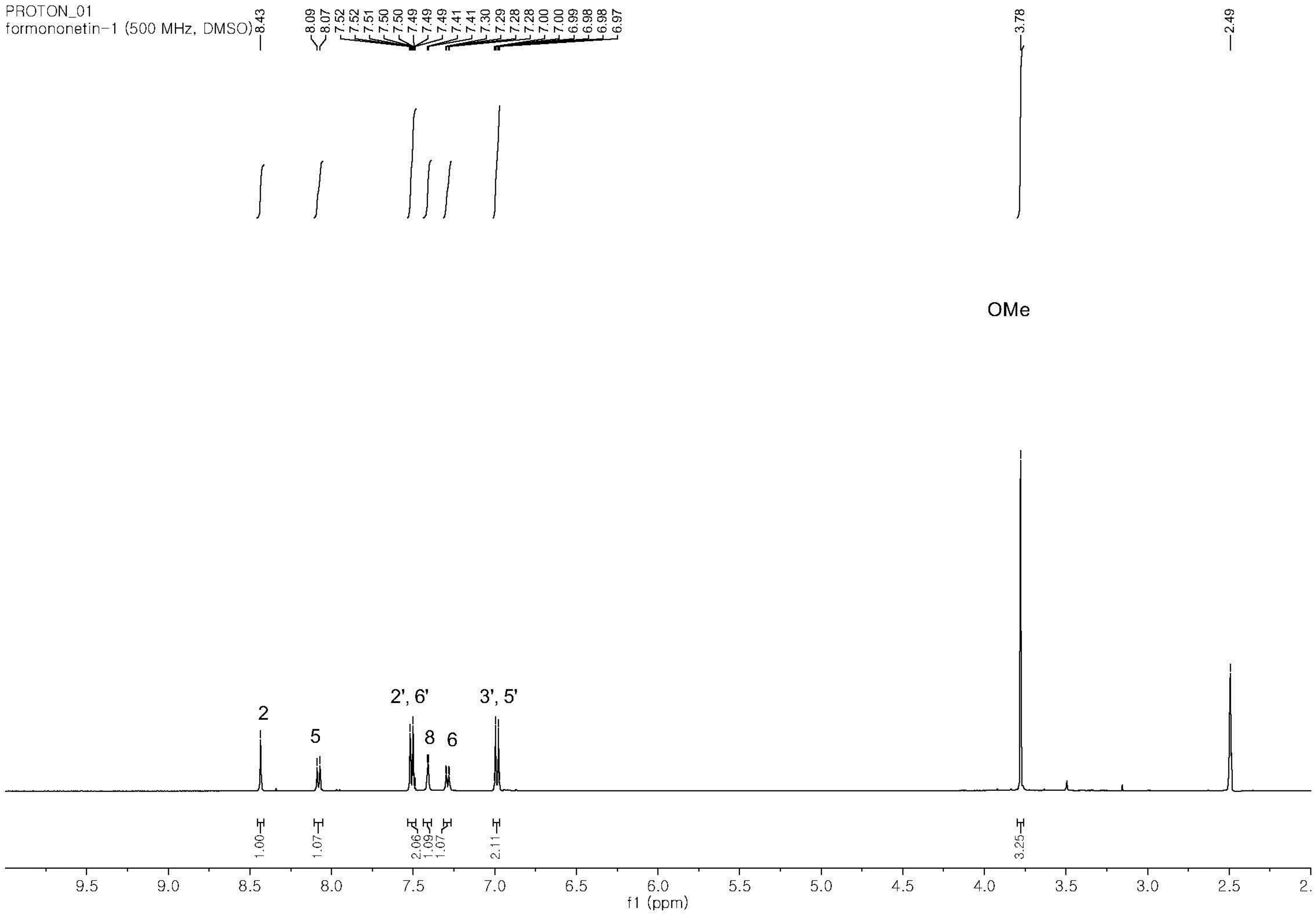

Figure S1. ^{1}H NMR of formononetin 7-*O*-phosphate (FMP, 1)

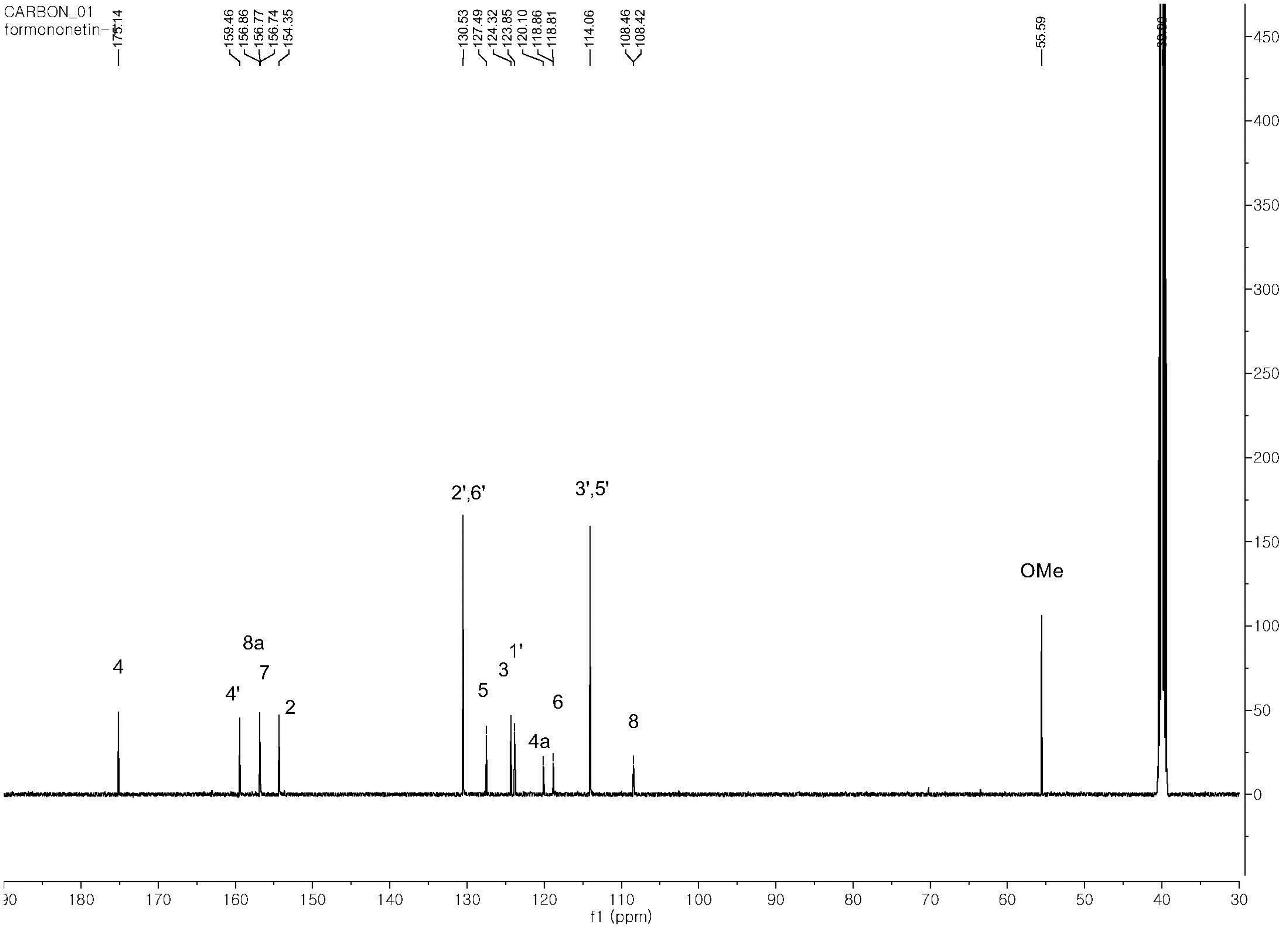

Figure S2. ^{13}C NMR of formononetin 7-*O*-phosphate (FMP, 1)

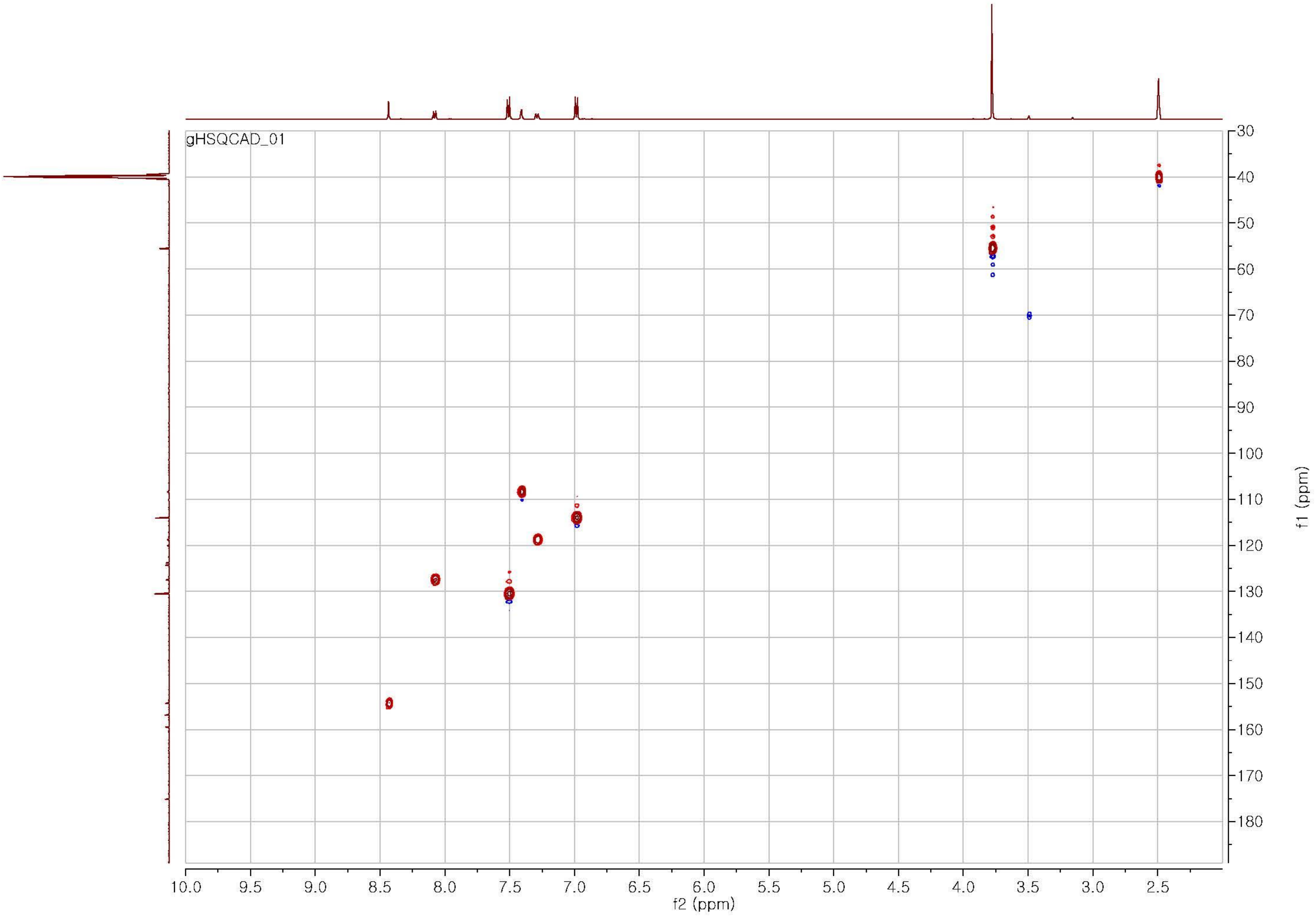

Figure S3. HSQC correlation of formononetin 7-*O*-phosphate (FMP, 1)

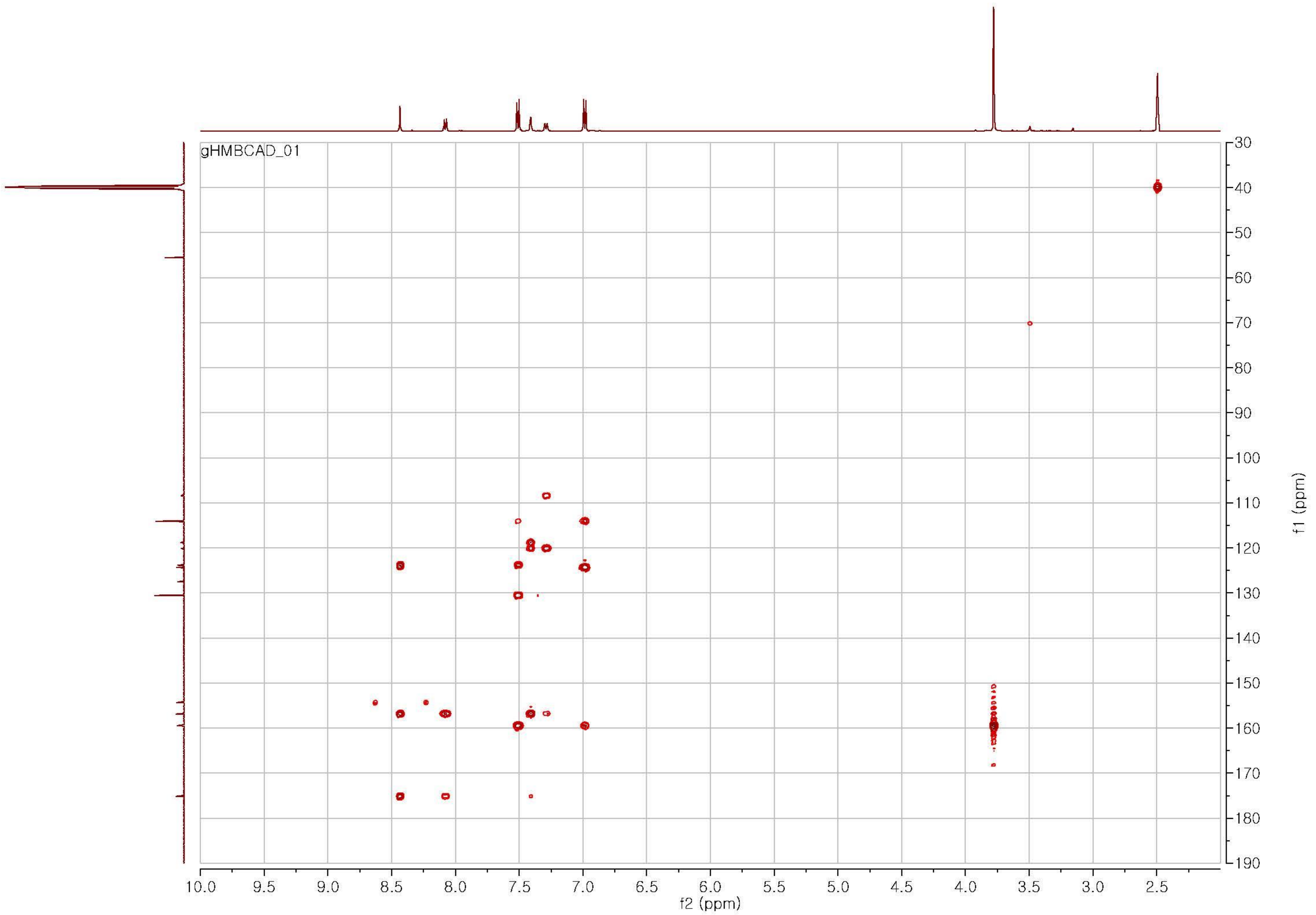

Figure S4. HMBC correlation of formononetin 7-*O*-phosphate (FMP, 1)

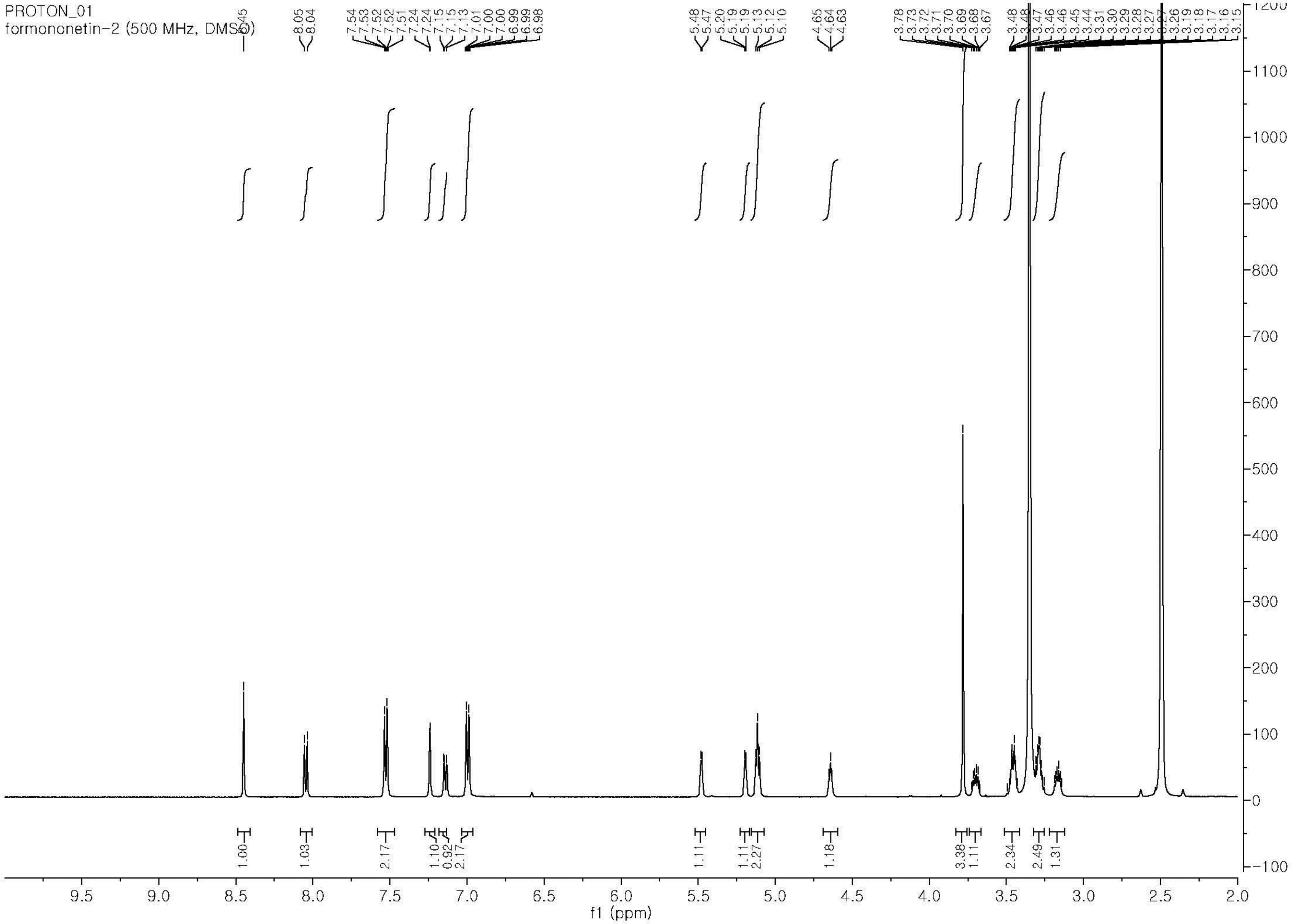

Figure S5. ^{1}H NMR of formononetin 7-*O*-glucoside
